# Advancing the Diagnosis of Non‐Hodgkin Lymphoma Through Next‐Generation Sequencing in Developing Countries: An Evaluation of Progress—A Narrative Review

**DOI:** 10.1002/hsr2.71873

**Published:** 2026-04-11

**Authors:** Mohammadreza Saeidnia, Mehdi Shokri, Hassan Nourmohammadi, Safa Radmehr, Mashallah Babashahi, Mohamad Moradi, Maryam Karimian, Mohammad Panji, Elahe Motevaseli

**Affiliations:** ^1^ Department of Hematology, School of Paramedical Shiraz University of Medical Sciences Shiraz Iran; ^2^ Department of Pediatrics, School of Medicine, Imam Khomeini Hospital Ilam University of Medical Sciences Ilam Iran; ^3^ Department of Internal Medicine, Razi Hospital Ilam University of Medical Sciences Ilam Iran; ^4^ Thalassemia & Hemoglobinopathy Research Center, Health Research Institute, Ahvaz Jundishapur University of Medical Sciences Ahvaz Iran; ^5^ Department of Pathobiology, School of Medicine Ilam University of Medical Sciences Ilam Iran; ^6^ Brigham and Women's Hospital Harvard Medical School Brigham and Women's Hospital. Boston Boston Massachusetts USA; ^7^ Department of Molecular Medicine School of Advance Technologies in Medical Tehran University of Medical Sciences Tehran Iran

**Keywords:** developing countries, diagnosis, next‐generation sequencing, NHL

## Abstract

**Background and Aims:**

Non‐Hodgkin lymphoma (NHL) is the most prevalent hematological malignancy worldwide and accounts for approximately 3% of all cancer cases and fatalities. Next‐generation sequencing (NGS) has advanced molecular diagnosis and targeted treatment in developed countries. However, developing countries face barriers like limited infrastructure, funding, and expertise, hindering wide NGS adoption. This narrative review evaluates the progress, challenges, and feasibility of using NGS for NHL diagnosis in developing countries.

**Methods:**

A comprehensive narrative literature review was conducted. We searched the PubMed, Scopus, and Google Scholar databases for relevant English‐language articles published between January 2008 and December 2025. The focus was on synthesizing evidence from studies applying NGS for NHL diagnosis, with particular emphasis on data from developing countries and comparisons with advancements in developed regions.

**Results:**

NGS has improved NHL subclassification accuracy and identified clinically relevant mutations, enabling personalized therapies. Studies from China, India, and South Africa demonstrate successful implementation of panel‐based NGS strategies. Despite this, challenges persist, including high costs, lack of standardized protocols, infrastructural deficits, and workforce shortages, limiting broader utilization in resource‐limited settings. Collaborative efforts and investments have begun to address these issues in some developing countries.

**Conclusion:**

NGS promises significant benefits in NHL diagnosis and management in developing countries. Overcoming financial, technical, and training barriers through targeted policies, funding, and international cooperation is crucial to harnessing its full potential. With ongoing advancements, NGS is poised to become a crucial tool for diagnosis and guiding therapy worldwide, including in resource‐limited settings.

**Abbreviations**
AITL: angioimmunoblastic T‐cell Lymphoma
BL: Burkitt lymphoma
ctDNA: circulating tumor DNA
DLBCL: diffuse large B‐cell lymphoma
FL52: signature (a 52‐gene expression profile associated with follicular lymphoma subtypes)
GEP: gene expression profiling
MCL: mantle cell lymphoma
MRD: minimal residual disease
NGS: next‐generation sequencing
NHL: non‐Hodgkin lymphoma
R&D: research and development
WES: whole exome sequencing
WHO: World Health Organization

## 1 Introduction

### 1.1 Importance of Accurate Diagnosis of Non‐Hodgkin Lymphoma (NHL)

Malignant lymphomas are a group of tumors with varying degrees of malignancy. They originate from lymphoid lineage cells, primarily B, T, or natural killer (NK) lymphocytes, at different stages of differentiation [1]. These cancers are classified as either Hodgkin's disease or non‐Hodgkin's lymphoma (NHL) [2]. NHL is more likely to spread to extranodal areas than Hodgkin's, with approximately 25% originating in these locations [3]. NHL stands out as the most prevalent hematological malignancy globally. Accounting for approximately 3% of cancer cases and deaths, the disease is more prevalent among men, individuals over 65, and those with autoimmune disorders or a family history of hematological cancers [4]. These disorders, known as mature lymphoproliferative disorders, arise from the malignant transformation and clonal expansion of lymphocytes in secondary lymphoid organs [5]. Among these disorders, the great majority of NHL subtypes are of B‐cell origin, commonly linked to defects in immunogenetic mechanisms that are active during germinal center B cell reactions [6]. For accurate diagnosis and standardized categorization of these heterogeneous disorders, the World Health Organization (WHO) classification system for lymphoid neoplasms is routinely employed [2]. Table 1 displays the classification of NHL based on the WHO criteria [7, 8]. Lymphoma treatment can be challenging due to uncertainties in presentation and potential complications [9]. However, the future of lymphoma treatment has rapidly evolved, and advances in understanding the molecular processes within lymphoma cells have facilitated the development of targeted therapies [10]. These approaches reduce the risk of adverse effects while effectively targeting lymphoma cells. Furthermore, improved diagnostic techniques and the identification of molecular markers have enhanced the availability of targeted therapies for patients [10]. Therefore, an accurate diagnosis, careful staging of the disease, and identification of adverse prognostic factors are fundamental to treatment selection [11].

**Table 1:** World Health Organization classification for NHL [7, 8].

**The WHO classification subtypes for NHL precursors**
Precursor B–lymphoblastic leukemia/lymphoma
Precursor T–lymphoblastic lymphoma/leukemia
**The WHO classification subtypes for peripheral B‐cell neoplasms**
B‐cell chronic lymphocytic leukemia/small lymphocytic lymphoma
B‐cell prolymphocytic leukemia
Lymphoplasmacytic lymphoma/immunocytoma
Mantle cell lymphoma
Follicular lymphoma
Extranodal marginal zone B‐cell lymphoma of (MALT) type
Nodal marginal zone B‐cell lymphoma ( ± monocytoid B cells)
Splenic marginal zone lymphoma ( ± villous lymphocytes)
Hairy cell leukemia
Plasmacytoma/plasma cell myeloma
DLBCL
Burkitt lymphoma
**The WHO classification subtypes for peripheral T‐cell and NK‐cell neoplasms**
T‐cell chronic lymphocytic leukemia/prolymphocytic leukemia
T‐cell granular lymphocytic leukemia
Mycosis fungoides/Sézary syndrome
Peripheral T‐cell lymphoma, not otherwise characterized
Hepatosplenic gamma/delta T‐cell lymphoma
Subcutaneous panniculitis‐like T‐cell lymphoma
Angioimmunoblastic T‐cell lymphoma
Extranodal T‐/NK‐cell lymphoma, nasal type
Enteropathy‐type intestinal T‐cell lymphoma
Adult T‐cell lymphoma/leukemia (HTLV) 1+
Anaplastic large cell lymphoma, primary systemic type
Anaplastic large cell lymphoma, primary cutaneous type
Aggressive NK‐cell leukemia

Abbreviations: DLBCL, diffuse large B‐cell lymphoma; HTLV, human T‐lymphotropic virus; MALT, mucosa‐associated lymphoid tissue; NHL, non‐Hodgkin lymphoma; WHO, World Health Organization.

Traditionally, the detection of these crucial molecular genetic alterations in hematologic malignancies has relied on conventional techniques such as cytogenetics, fluorescence in situ hybridization (FISH), or PCR assays [12]. While karyotyping remains the gold standard, its clinical utility is frequently limited by low chromosomal resolution. FISH, despite being a more accessible alternative, presents several significant limitations. These include poor sensitivity for detecting low‐level clones in minimal residual disease (MRD), inefficiency in identifying unbalanced translocations, and a lack of standardized cutoff values across different probes. Consequently, even when cutoffs are statistically defined, the prognostic relevance of low‐level abnormalities often remains uncertain, underscoring the need for careful interpretation of FISH results [13].

In contrast, NGS overcomes many of these challenges by allowing the simultaneous detection of both dominant and subclonal genetic markers. This high‐throughput capability not only enhances cost‐effectiveness but also substantially reduces turnaround time compared to sequential single‐assay testing. As a result, NGS has become a cornerstone for comprehensive molecular profiling in hematologic malignancies [12]. Since 2016, numerous studies have highlighted the expanding application of NGS in hematologic malignancies, including multiple myeloma (MM), primarily focusing on sequencing at both diagnosis and relapse to delineate the genetic evolution of the disease [13].

In summary, by overcoming the key limitations of conventional diagnostic techniques, NGS provides critical added value through comprehensive genomic profiling. It has become an indispensable tool for precise diagnosis, risk stratification, and guiding targeted therapy in modern hematologic oncology.

Therefore, the purpose of this study is to comprehensively review recent developments in the use of NGS for the diagnosis of NHL, with a particular focus on its progress, ongoing challenges, and the practical feasibility of implementing this transformative technology in developing countries.

## 2 Methods

This narrative review aimed to examine recent developments in the application of next‐generation sequencing (NGS) for the diagnosis of non‐Hodgkin lymphoma (NHL), with particular emphasis on its progress, current challenges, and feasibility in developing countries. A structured literature search was conducted in PubMed, Scopus, and Google Scholar for English‐language articles published between January 2008 and December 2025. The search strategy combined keywords and Medical Subject Headings (MeSH) terms, including “NHL,” “next‐generation sequencing,” “high‐throughput sequencing,” “diagnosis,” “developing countries,” “resource‐limited settings,” and selected lymphoma subtypes (e.g., “diffuse large B‐cell lymphoma [DLBCL]” and “follicular lymphoma”).

We included original research articles, case series, and review studies that reported on the diagnostic use of NGS in NHL. Selection criteria prioritized studies relevant to resource‐limited or developing settings, while evidence from developed countries was also reviewed to provide a comparative perspective. Extracted studies were analyzed thematically to identify major technological advances, persistent diagnostic and implementation barriers, and emerging strategies for integrating NGS into routine diagnostic workflows in resource‐constrained environments.

## 3 Diagnostic Challenges and Advances in Non‐Hodgkin Lymphoma in Developed and Developing Countries

### 3.1 Advancements and Remaining Challenges in NHL Diagnosis in Developed Countries

In developed countries, the diagnostic approach to NHL is grounded in the 2008 WHO Classification, which emphasizes the integration of morphological findings with clinical features and often requires complex ancillary studies for accurate diagnosis. Despite these advancements, lymphoma pathology remains complex and rapidly evolving, requiring frequent updates to classification criteria and diagnostic methods. Reflecting this progress, the frequency of misclassified NHL cases in these regions is significantly lower (2.2%) compared to the developing world (7.5%), indicating a higher degree of diagnostic precision [14].

Epidemiological data from North America further illustrate distinctive disease patterns, showing that follicular lymphoma (FL) has the highest incidence among whites. Moreover, studies highlight that US‐born Asians exhibit a higher incidence of FL than foreign‐born Asians, underscoring the influence of environmental and lifestyle factors, including dietary patterns such as higher intake of red meat and saturated fat [14]. These lifestyle differences may partly contribute to the higher prevalence of FL in developed regions compared with areas characterized by different dietary patterns [14, 15]. In addition, the overall median age of patients with both low‐grade (LG) and high‐grade (HG) B‐cell NHL is significantly higher in developed countries than in developing regions, further differentiating the patient profiles between these settings [14].

Although the WHO classification framework for NHL was first introduced in 2001, its subsequent revisions and their consistent implementation in routine clinical practice remain underexplored and warrant further evaluation [16]. Based on this established diagnostic framework, NGS has evolved from a research tool to a key part of the routine clinical workflow for NHL in developed countries. For example, the use of a standardized 59‐gene NGS panel in a consecutive patient cohort resulted in a high mutation detection rate (94%). This directly aided in molecular subclassification and identified potential biomarkers for monitoring and therapy [17]. This reflects a phase of “innovation consolidation,” where the primary application of NGS is to refine diagnosis within established classification systems (such as the WHO guidelines), optimize risk stratification, and guide targeted treatment decisions [12]. Consequently, in these settings, NGS is firmly established as a core component of modern hematologic malignancy management [12].

### 3.2 Diagnostic Challenges in Developing Countries

A major challenge in diagnosing NHL is its geographic variability, with significant differences between developing and developed regions. Developing countries exhibit a lower overall proportion of B‐cell NHL and higher rates of T‐cell and NK‐cell NHL compared with developed regions [14]. Nevertheless, among B‐cell lymphomas, DLBCL remains the most frequent subtype in the developing world, whereas follicular lymphoma (FL) is less prevalent [14]. These variations underscore the necessity for region‐specific diagnostic criteria and training programs tailored to local healthcare contexts. Lifestyle factors, along with the host's genetic makeup, also play a crucial role in the development of NHL [14].

However, addressing region‐specific variations in NHL is complicated by significant diagnostic challenges in developing countries. In these regions, NHL diagnosis is often hindered by a lack of trained pathologists, limited access to essential diagnostic technologies, and the absence of immunohistochemistry methods, which complicates tissue diagnosis. Studies show that many pathologists in developing countries lack specialized training in hematopathology and access to critical diagnostic studies [14, 18].

Furthermore, these challenges are exacerbated by a higher incidence of unclassifiable NHL cases due to substantial obstacles related to specimen handling and tissue processing. These issues frequently result in low‐quality slides that complicate accurate diagnoses [19]. Studies indicate a notably higher misclassification rate in developing regions compared to developed countries, which underscores the pressing need for improved diagnostic infrastructure and training [14]. Additionally, patients from these regions tend to be younger at diagnosis for both low‐grade (LG) and high‐grade (HG) B‐NHL compared to those in developed countries [14]. This further emphasizes the need for structured quality‐assurance programs and standardized diagnostic pathways tailored to resource‐limited settings.

Accurate staging is another challenge in developing countries that is crucial for determining appropriate therapy. In high‐income countries, precise staging informs treatment assignment; for instance, limited‐stage disease that is amenable to complete surgical excision (considered low risk) may require minimal therapy, while involvement of the bone marrow and/or central nervous system (considered high risk) necessitates more intensive treatment. However, risk‐adapted treatment stratification is often difficult to implement in many developing countries because of limited access to staging procedures, diagnostic imaging, and overall healthcare capacity [18]. Patients in developing countries often present with co‐morbidities such as active infections and malnutrition, which complicate their treatment. Moreover, late presentations can lead to severe complications like tumor lysis syndrome, a condition that is particularly challenging to manage due to limited access to necessary medical resources. This limitation ultimately contributes to poorer outcomes for patients [20, 21].

Furthermore, these disparities extend beyond clinical presentation; studies on gender distribution reveal a significantly higher incidence of NHL in men compared to women within these regions. This disparity has been partly attributed to gender inequalities in access to healthcare in some developing countries [14]. Women may have less access to medical care or may be less inclined to seek it, resulting in underdiagnosis of lymphoma among this population [22]. Consequently, diagnosing NHL in developing countries poses multiple challenges that necessitate targeted interventions and improvements in healthcare infrastructure.

In this context, emerging molecular approaches are increasingly being explored as potential tools to mitigate some of these diagnostic limitations. To address these challenges, pioneering studies in resource‐limited settings use targeted NGS panels to profile NHL, identifying both recurrent and novel mutations and highlighting molecular heterogeneity [23]. However, these efforts remain largely research initiatives and face the fundamental challenge of limited resources, which restricts the wide availability of essential genomic sequencing technologies in these settings [24]. Thus, the primary aim shifts from clinical integration to proving viability and building foundational genomic capacity.

The diagnostic landscape for NHL thus reveals a fundamental dichotomy. In developed countries, NGS is a tool for refining precision within mature systems (“innovation consolidation”). In developing nations, it is a pioneering technology for building basic genomic capacity amidst foundational constraints. The disparity lies not in access alone, but in the stage of diagnostic maturity. Closing this gap requires moving beyond equipment transfer to integrated strategies that simultaneously strengthen core pathology and develop sustainable, context‐specific genomic pathways.

## 4 Overview of Next‐Generation Sequencing (NGS) Technology

### 4.1 Principles of NGS

NGS enables the simultaneous sequencing of multiple DNA or RNA fragments. Compared with traditional Sanger sequencing, NGS is significantly faster, generates larger data volumes, and is more cost‐effective [25]. Moreover, it integrates diverse chemistries, sequencing platforms, and bioinformatics tools to enable efficient genetic analysis [26]. This technological capability has profoundly advanced the understanding of somatic diseases, particularly in oncology and hematologic malignancies [27].

Specifically in non‐Hodgkin lymphoma (NHL), NGS enables precise molecular subtyping by identifying genetic markers characteristic of distinct lymphoma entities. This molecular characterization provides an integrated diagnostic framework that complements conventional morphology and immunophenotyping, facilitating more accurate disease classification. In addition, the comprehensive genomic profiling achieved by NGS enhances the understanding of the molecular architecture of NHL and supports risk stratification at the time of diagnosis [27, 28]. This comprehensive genomic analysis is enabled by a streamlined core workflow comprising DNA fragmentation, library preparation, massively parallel sequencing, bioinformatics analysis, and variant annotation [26].

### 4.2 Key Steps in Next‐Generation Sequencing (NGS) Workflow

The NGS workflow comprises several critical steps that together enable massively parallel sequencing. Target DNA is fragmented into short segments (100–300 bp) via mechanical shearing, enzymatic digestion, or sonication; these fragments are then isolated via hybridization capture or PCR amplification [29, 30, 31, 32]. Library preparation follows, during which sequencing adapters and sample‐specific indices are added to enable multiplexing and attachment to the sequencing platform [26]. Sequencing is performed on specialized platforms like Illumina or Ion Torrent [26]. Subsequent bioinformatics analysis includes base calling, read alignment, variant identification, and annotation against a reference genome [26]. This integrated process has transformed genomic research [33, 34] and has enabled the expansion of NGS applications into various omics fields [35].

**Figure 1:**
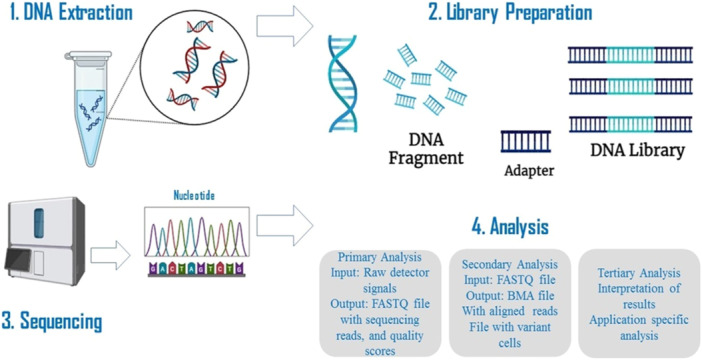
Overview of the steps involved in the next‐generation sequencing (NGS) method. This figure highlights the key stages of NGS, including sample preparation, library preparation, sequencing, and data analysis. It is important to note that various NGS methods, such as whole genome sequencing (WGS), whole exome sequencing (WES), and RNA Sequencing (RNA‐Seq), each have distinct workflows and applications.

In hematological malignancies, NGS provides a cost‐effective approach to comprehensive genetic profiling and enhances the characterization of disease‐associated genomic alterations; however, it also poses challenges related to data interpretation and workflow optimization [36]. This is particularly relevant in NHL, where NGS enables the systematic identification of recurrent somatic alterations that support molecular classification and diagnostic stratification [28]. Figure 1 illustrates an overview of the NGS process.

### 4.3 Applications of NGS Assays

NGS platforms employ various high‐throughput approaches whose methodological differences affect sequencing quality, yield, and specific applications [37]. Among NGS assays, whole‐exome sequencing (WES) targets all protein‐coding regions ( ~ 1% of the genome) and is primarily a research tool [26]. Additionally, RNA sequencing (RNA‐Seq) applies NGS to analyze the transcriptome of a cell, primarily focusing on mRNA and other longer non‐coding RNAs. A specialized application of this technique focuses on mRNA to identify clinically significant fusion genes, particularly relevant in oncology [26]. In clinical cancer diagnostics, targeted panel sequencing has become the predominant NGS assay; by concentrating on dozens to hundreds of disease‐specific genes, it achieves greater sequencing depth and more sensitive mutation detection compared to broader approaches such as whole‐genome sequencing (WGS) [26]. In NHL, this approach supports standardized molecular classification and diagnostic risk stratification, reinforcing the transition from purely morphology‐based assessment toward integrated genomic diagnostics.

### 4.4 Comparative Performance of NGS Platforms

Comparative studies indicate that the choice of NGS platform significantly influences sequencing cost, coverage, and accuracy, which is particularly relevant for implementing NGS in routine lymphoma diagnostics. Direct comparisons between Illumina MiSeq and Ion Torrent platforms have shown that platform selection and library preparation strategies affect genome coverage and consensus sequence accuracy. Ion Torrent S5 510 chip runs generated more reads at a lower cost per sample than Ion Torrent PGM 318 runs, although insertions and deletions in homopolymer regions reduced consensus accuracy. In lower‐throughput sequencing, the Illumina MiSeq platform demonstrated a lower cost per sample, while in higher‐throughput runs, cost differences between the two platforms were reduced. Overall, both Illumina MiSeq and Ion Torrent S5 platforms represent viable sequencing options, each with specific advantages and tradeoffs [38]. Additional analyzes reported near‐complete coverage for GC‐rich and moderately AT‐rich genomes using both platforms; however, substantial coverage bias was observed for extremely AT‐rich genomes with the Ion Torrent PGM platform. Although Ion Torrent data yielded slightly higher variant detection rates, this was accompanied by an increased false‐positive rate, highlighting platform‐specific differences in data quality and application suitability [39].

Collectively, these findings demonstrate that both Illumina MiSeq and Ion Torrent platforms generate usable sequencing data. Although these comparative studies were not conducted in developing countries, their reported differences in cost, throughput, and error profiles provide relevant insights for NGS platform selection in resource‐limited settings.

### 4.5 Applications of NGS in Hematological Malignancies

The molecular characterization of hematologic malignancies has traditionally relied on conventional techniques like cytogenetics, fluorescence in situ hybridization (FISH), and PCR [12]. Assays such as digital droplet PCR (ddPCR) offer high precision for quantifying specific mutations [40], while FISH is invaluable for identifying translocations and copy‐number alterations that define risk categories [41]. Collectively, these tools are integral to clinical management [42]. In recent years, however, high‐throughput, massively parallel NGS has transformed the field, providing unprecedented insights into disease biology, progression, and therapeutic response [43]. By enabling simultaneous detection of numerous dominant and subclonal markers, NGS improves cost‐effectiveness and reduces turnaround time compared to sequential single‐assay testing [12]. Consequently, NGS has become indispensable for characterizing hematologic malignancies across the clinical spectrum, with applications in diagnosis, prognostication, and post‐treatment monitoring [12]. Its evolution from a research tool to a standard in clinical trials underscores its dual role in diagnosis and guiding therapy [44]. The adoption of targeted DNA‐based NGS panels is now widespread in clinical laboratories, a trend reinforced by the WHO's 2022 classification, which emphasizes genetic subtyping [12, 45]. Complementing this, targeted RNA‐based NGS is routinely used to detect fusion transcripts in accordance with WHO guidelines [12]. Given its analytical sensitivity at approximately 1%–2% variant allele frequency and its cost‐effectiveness, targeted NGS is considered optimal for routine testing in conditions such as AML [45].

Non‐targeted NGS approaches, such as whole‐genome sequencing (WGS), have also proven valuable for comprehensive genomic profiling in hematologic malignancies. For instance, WGS in acute myeloid leukemia (AML) has replicated all cytogenetically detectable translocations and copy‐number alterations [46], while in pediatric acute lymphoblastic leukemia (ALL), it has consistently identified subtype‐defining lesions and revealed novel variants, including MAP kinase pathway fusions [47].

The co‐occurrence of CD79B and MYD88 mutations defines a distinct molecular subgroup within DLBCL, characterized by activated B‐cell (ABC) origin, frequent extranodal involvement, and mutual exclusivity with high‐grade double/triple‐hit lymphomas. This genetic profile is implicated in pathogenesis and may drive treatment resistance. Clinically, this subgroup exhibits the poorest prognosis with standard chemoimmunotherapy but shows promising responses to targeted agents such as Bruton's tyrosine kinase (BTK) inhibitors (e.g., ibrutinib), highlighting the BCR pathway's central role. Thus, the detection of these mutations by NGS directly informs clinical decision‐making: it identifies a patient subgroup with poor prognosis on standard therapy who are prime candidates for targeted treatment with BTK inhibitors such as ibrutinib. This exemplifies how molecular findings translate into personalized therapeutic strategies, although optimal strategies for single‐mutant cases require further study [48].

Thus, NGS technologies are pivotal for delineating the molecular architecture of hematologic malignancies throughout their course. To fully realize this potential, however, broader implementation requires enhanced laboratory infrastructure and bioinformatics capabilities [12].

## 5 NGS‐Based Approaches for NHL Diagnosis

### 5.1 Gene Expression Profiling

Recently, low‐throughput quantitative RNA assays have shown their effectiveness for routine classification of high‐grade B‐cell non‐Hodgkin lymphoma (B‐NHLs) [49]. Conversely, approaches bypass the complexities of pan‐genomic gene expression profiling (GEP), which is rarely feasible in routine diagnostic settings, by targeting a limited set of genes associated with well‐defined gene expression signatures [49]. Notably, these assays only capture a limited aspect of the complexity of B‐NHLs, which restricts their effectiveness in clinical practice [49]. In this context, a crucial goal of GEP is to identify oncogenic pathways that are differentially implicated in newly discovered subsets of lymphoid malignancies, providing potential targets for future drug development [50]. Accordingly, researchers utilized NanoString expression profiling to classify NHL cases, enabling accurate tumor characterization and supporting targeted therapies [51]. Furthermore, GEP using microarrays has enhanced our understanding of tumor biology, revealing how molecular differences can affect therapy responses and patient outcomes [52]. In DLBCL, GEP has identified at least two distinct diseases with differing responses to chemotherapy and oncogenic pathway inhibition. In follicular lymphoma, this technique has demonstrated that the host immune response plays a crucial role in determining patient outcomes and can significantly predict survival at diagnosis. Consequently, immunologic therapies targeting the host immune response hold great promise for improving survival rates in FL patients [52]. In another study, researchers developed a transcriptomic classifier using GEP to enhance the accuracy of peripheral T‐cell lymphoma (PTCL) diagnosis. This approach demonstrated high sensitivity, specificity, and precision, making it a reliable tool for integration into routine clinical practice [53]. Moreover, alongside GEP, NGS has contributed to identifying distinct clinicobiological entities and refining lymphoma classification, particularly in PTCL [8]. These advancements highlight the complementary roles of GEP and NGS in enhancing our understanding of lymphoma biology and improving disease categorization. It is noteworthy that, the use of GEP in NHL offers valuable prognostic insights at diagnosis and can lead to therapeutic strategies that improve patient outcomes [52].

### 5.2 Targeted Sequencing of NHL‐Associated Genes

B‐NHL includes several clinically and phenotypically distinct subtypes, each with unique molecular etiologies [54]. Cooperative interactions among co‐occurring genetic alterations are frequently observed in cancer, and this emerging field warrants further investigation [55]. Recent data suggest that some of these genetic alterations may possess prognostic and predictive significance [56]. However, extensive genetic analyzes, such as whole exome or large panel sequencing, remain costly and time‐consuming, thereby limiting their routine diagnostic application [56]. Importantly, common subtypes of B‐NHL, such as DLBCL, have been thoroughly examined at the genomic level, but rarer subtypes, like mantle cell lymphoma, remain less well characterized [54]. A cross‐sectional genomic profiling study focused on a large group of tumors, identifying genes and functional characteristics frequently altered by genetic changes in B‐NHL. The results revealed that mutations in genes involved in epigenetic control and gene expression transcription, known as the FL52 signature (a 52‐gene expression profile associated with follicular lymphoma subtypes), were present in 96% of FL tumors, with one or more mutations occurring in this category. Moreover, mutations in these genes were also observed in the majority of Burkitt lymphoma (BL), DLBCL, and mantle cell lymphoma (MCL) tumors, underscoring the conservation of this functional marker across B‐NHL subtypes [54]. It is noteworthy that, the genetic deregulation of epigenetic mechanisms, the ubiquitin‐proteasome system, and transcriptional control of gene expression should be regarded as a general hallmark of B‐NHL [54]. Furthermore, the examination of the studies indicates that genetic investigation and targeted sequencing have become essential to lymphoma diagnosis, enabling improved classification of lymphoma, patient risk assessment, and treatment response prediction [57].

### 5.3 Whole‐Exome and Whole‐Genome Sequencing for NHL

The diagnosis for NHLs includes morphological and immuno‐phenotypic examinations, as well as chromosomal and molecular analyzes. Among them, NGS methods have supplied valuable Supporting data in diagnosis, prognosis, and treatment [8]. NGS identifies many tumor mutations or DNA sequences (IGH or TCR loci) at the same time, overcoming the constraints of conventional methods that only monitor a single mutation or clone. Several lymphoma subtypes have shown that NGS‐based circulating tumor DNA (ctDNA) genotyping is both feasible and reliable for diagnosis and prognosis [58, 59, 60, 61]. NGS‐based analysis of ctDNA has emerged as a promising method for identifying genetic patterns across various lymphoma subgroups, indicating that ctDNA could serve as a noninvasive and practical biomarker for diagnosis [60]. This advancement complements the significant progress made through exome sequencing (ES), which has transformed disease research by focusing on protein‐coding regions of the genome and facilitating efficient data generation and analysis [62]. Recent studies have employed whole exome sequencing (WES) to analyze various primary immunomorphological subtypes of lymphomas, including DLBCL, BL, Follicular lymphoma, Mantle cell lymphoma, Splenic marginal zone lymphoma, and PTCL [63]. In this direction, in a 2017 study, Reddy et al. conducted whole‐exome sequencing (WES) on tumors from 1001 DLBCL patients to identify genetic drivers and their clinical significance. The analysis revealed 150 genes involved in DLBCL pathogenesis, categorized into four groups: signaling pathway genes (e.g., MTOR, PIK3R1), transcription and translation genes (e.g., SF3B1), B‐cell differentiation genes (e.g., EBF1), and cell growth/proliferation genes (e.g., MYC). Key mutations included MYD88 in the ABC subtype and XPO1 in the GCB subtype [27, 64]. Of course, another study identified four primary genetic subtypes of non‐GCB DLBCL—MCD, BN2, N1, and A53—using the LymphGen algorithm [65]. Additionally, Schmitz et al. a comprehensive genomic analysis was conducted on 574 DLBCL patients using whole‐exome and transcriptome sequencing, DNA copy number analysis, and deep targeted sequencing. This research identified four distinct subtypes of DLBCL: MCD, characterized by MYD88L265P and CD79B mutations; BN2, defined by BCL6 fusions and NOTCH2 mutations; N1, associated with NOTCH1 mutations; and EZB, noted for EZH2 mutations and BCL2 translocations [27, 66]. Overall, examining the results of various studies reveals that exome sequencing serves as a powerful tool for identifying novel variants and understanding familial disease predisposition [67]. The increasing availability of NGS technologies, including whole genome sequencing WGS and WES, has expanded their applications beyond cancer research to clinical settings [68]. Although NGS data in lymphomas require further validation before routine clinical use, it is reasonable to anticipate that their clinical application is imminent, at least in part [69]. The current recommendations do not provide specific guidance on the sequencing methods utilized for the diagnosis and prognosis of NHL. Currently, there is no standardized approach, and various factors such as gene selection, sequencing platform, read depth, and variant analysis may vary among laboratories. Therefore, it is imperative to establish panel standardization, particularly for NGS panels focusing on the lymphoid lineage, to ensure their widespread availability for clinical applications [27].

## 6 Progress in NGS‐Based NHL Diagnosis in Developing Countries

### 6.1 Current Status and Challenges in Developing Countries

Health, education, and scientific research are critical in developing countries, as they are closely tied to national well‐being. Genomics and other “omics” sciences are valuable tools with applications across medicine, agriculture, and healthcare. The ability to acquire expertise and utilize “omics” technologies empowers developing countries to advance knowledge and enhance health risk identification, diagnosis, treatment, and prevention strategies [24]. However, recently, it has been shown that NGS is one of the most powerful and efficient diagnostic tools for rare Mendelian disorders, especially those that exhibit genetic diversity [70]. Subsequently, numerous NGS technologies have been introduced, bringing about a remarkable revolution in the extraction of genetic data at an affordable cost and in large volumes. NGS has empowered scientists to expand the applications of genome sequencing to unprecedented levels and create innovative applications across various fields [71]. Developing countries often face low human development, limited research funding, and scarce resources, which can hinder genomic research. Access to essential genome sequencing technologies may also be limited due to cost, infrastructure, and expertise [24]. Indeed, despite the notable advancements in cost reductions associated with NGS technology, establishing a genome sequencing laboratory with NGS capabilities can still present challenges, particularly in developing countries [71]. Setting up NGS facilities in developing countries can be particularly costly due to factors such as export costs, customs duties, and local businesses' profit margins. The financial burden associated with establishing and maintaining these facilities often exceeds the funding capabilities of many scientists in these regions [24]. Therefore, this level of expense significantly surpasses the available funding for scientists in most developing countries [24]. Of course, some developing countries, such as India, South Africa, Mexico, and Brazil, have advanced genomic applications through funding, institutional development, and personnel training. However, many regions, particularly in Africa, still face significant barriers [24]. Hence, addressing these challenges and improving access to genomic technologies in developing nations is essential for advancing healthcare and research in these regions.

### 6.2 Case Studies Demonstrating the Utility of NGS in NHL Diagnosis

Recent NGS‐based studies have significantly enhanced our understanding of the genetic landscapes of B‐cell and T‐cell lymphomas, identifying genomic biomarkers that improve sub‐classification and diagnosis accuracy [28]. WES has provided the most comprehensive molecular assessment of lymphoma genetics by sequencing all coding sequences using high‐throughput NGS. This approach has been applied to major lymphoma subtypes such as DLBCL, BL, follicular lymphoma, mantle cell lymphoma, splenic marginal zone lymphoma, and PTCL [63]. NGS data have also revealed recurrent somatic mutations in NHL that can be targeted therapeutically to reduce resistance cases [27]. Building on these advancements, studies assessed the utilization of sequencing technologies in developing countries by examining data from the Genome Online Database (GOLD) and World Bank classifications. Developed countries have more sequencing centers, but nations like China, India, and Brazil are also contributing significantly due to higher R&D investments. However, limited funding remains a major barrier to fully leveraging genome sequencing in these regions [24]. However, among the series of case studies in developing countries regarding the use of gene sequencing techniques for cancer diagnosis, particularly NHL, a notable study was conducted in China. In this research, the genetic landscape was analyzed in 96 patients with DLBCL using a panel‐based NGS strategy. The study found that the most frequently mutated genes in patients were KMT2D (30%), PIM1 (26%), SOCS1 (24%), MYD88 (21%), and others. Notably, mutations in SPEN (17%) and DDX3X (6%) were more prevalent than in Western studies. Using the LymphGen algorithm, 34% of patients were genetically classified, with various subtypes identified. Additionally, MYD88 L265P, TP53, and BCL2 mutations were associated with poor prognosis in DLBCL. This study underscores the genetic heterogeneity of DLBCL and illustrates the role of panel‐based NGS in predicting prognosis and guiding potential molecular targeted therapies for the disease [72]. Additionally, another study conducted in Brazil demonstrated that molecular pathology techniques can provide significant benefits to individuals diagnosed with cancer. Hence, the panel suggests concrete and feasible recommendations for the integration of molecular pathology into cancer treatment in Brazil [73].

On the other hand, regarding the application of NGS in diagnosing NHL in developed countries, a notable study from Denmark can be referenced. This study utilized NGS to analyze 298 patients with newly diagnosed and relapsed/refractory NHL. The custom NGS panel focused on 59 genes and successfully identified mutations in 94% of the samples. While most lymphomas were definitively classified, 24 cases were categorized as small B‐cell lymphomas without specific features. The findings of this study demonstrated that NGS significantly enhances diagnostic accuracy and aids in identifying potential biomarkers for monitoring and treatment in NHL [17]. In another study conducted in Spain, researchers aimed to validate a NGS lymphoid panel for diagnosing common types of NHL, including FL and DLBCL. The study found that 372 somatic alterations were detected in 93.6% of patients, demonstrating the potential of NGS as a complementary tool in diagnosing these diseases [74]. Similarly, a study involving 108 patients with unclassifiable low‐grade B‐cell non‐Hodgkin lymphoma (LI B‐NHL) was conducted over 4 years at 14 UK centers. Utilizing a targeted NGS panel, researchers identified recurrent MYD88 mutations, particularly in patients with splenomegaly. These findings highlight NGS's potential to enhance diagnosis and facilitate access to new therapies, with ongoing 5‐year follow‐up data aimed at improving risk stratification and patient management [62]. Also, NGS analyzes of high‐grade B‐cell lymphoma (HGBL, NOS) reveal that this lymphoma exhibits heterogeneity and includes activated B‐cell lymphomas with mutations in MYD88, CD79B, or TBL1XR1 [75].

Nevertheless, the integration of NGS into clinical practice is expected to significantly advance the understanding and treatment of various lymphoma subtypes, ultimately leading to better patient outcomes and more personalized therapeutic approaches. Thus, it can be concluded that in the case of NHL, a deeper understanding of the mechanisms underlying tumorigenesis and proliferation, along with the identification of genetic markers specific to various lymphoma subtypes, has resulted in more accurate classification and diagnosis [27]. Table 2 presents the key findings related to the diagnosis of NHL using NGS techniques in studies conducted in both developing and developed countries.

**Table 2:** Key findings in the diagnosis of NHL using (NGS) techniques in studies conducted in developing and developed countries.

Study	Country	NHL subtype	Key findings
Cao et al. [72].	China	DLBCL	Genetic heterogeneity
			Role of panel‐based NGS in prognosis and targeted therapy
Morgan et al. [51]	Malawi	Burkitt lymphoma DLBCL	Advanced Diagnostics Improve NHL Subclassification
			Molecular Profiling Enables Targeted Therapy
Mamgain at al. [23]	India	DLBCL	Identification of targetable mutations in diffuse large B‐cell lymphoma subtypes
Breinholt, et al. [17].	Denmark	B‐NHL Subtypes	Mutation detection rate
			Classification of lymphomas
			Diagnostic value
Bastos et al. [74].	Spain	FL, DLBCL	Mutation detection
			Alterations in FL and DLBCL
			Correlation with disease stage
Matthew et al. [62].	UK	LI B‐NHL	Improve detection Facilitate access to new therapies

Abbreviations: DLBCL, diffuse large B‐cell lymphoma; FL, follicular lymphoma; LI B‐NHL, low‐grade B‐cell non‐Hodgkin lymphoma; NGS, next‐generation sequencing.

## 7 Potential Impact of NGS on NHL Management

### 7.1 Improved Risk Stratification

The study of gene expression profiles in various tumor types and subtypes enables the identification of additional markers associated with clinical outcomes, invasion risks, and metastasis. This approach not only refines existing classifications but also allows for the proposal of new ones based on the molecular characteristics of the tumors [27]. However, the significant progress made in recent years can be attributed to advancements in molecular genetics. These developments have facilitated a transition from analyzing individual genes and markers to conducting comprehensive studies on multiple genes or their expressed products simultaneously, particularly within the realm of cancer research [27].

### 7.2 Personalized Medicine Selection

While tumor genomic profiling for personalized therapy is increasingly being implemented in clinical practice, it is also undergoing more formal evaluation in clinical trials [76]. On the other hand, the knowledge gained from the genomic mapping of NHL will soon enable the targeting of molecular pathways responsible for treatment resistance, or conversely, the inhibition of pathways crucial for halting uncontrolled tumor proliferation [27]. Studies indicate that the co‐occurrence of mutations in MYD88 and CD79B can predict the response to Ibrutinib. This highlights the potential clinical utility of genomic profile data in DLBCL, which may significantly influence clinical practice [77]. Data obtained from genome sequencing in mantle cell lymphoma (MCL) have identified a subgroup of patients with inactivating mutations in the SWI‐SNF chromatin‐remodeling complex, which lead to BCL‐XL upregulation and subsequent resistance to the therapeutic combination of Ibrutinib and Venetoclax [78]. Many T‐cell lymphomas carry mutations in epigenetic regulatory genes, such as TET2, DNMT3A, and IDH2, with these mutations being most prevalent in angioimmunoblastic T‐cell lymphoma (AITL). Consequently, the use of HDAC inhibitors or demethylating agents may offer potential therapeutic benefits [27]. Overall, personalized medicine will not only target a single mutation using a specific drug. It can also identify molecular targets with synergistic co‐stimulatory or inhibitory effects, enhancing therapeutic efficacy [27]. Also, the complexities of genomic data and its clinical applications indicate that tumor molecular panels can provide valuable guidance to oncologists and patients seeking to effectively implement personalized, genetically targeted therapies [76].

### 7.3 Monitoring of Minimal Residual Disease

In hematological malignancies, minimal residual disease (MRD) refers to the presence of residual leukemic cells that remain undetected by conventional morphological methods [79]. In this context, MRD is a crucial parameter for guiding clinical management. Traditionally, real‐time quantitative PCR (qPCR) and flow cytometric technologies have been extensively employed for MRD monitoring [80]. NGS is a promising tool for MRD detection and has the potential to become the new gold standard for several hematological diseases in the near future. Indeed, standardization of the workflow is essential before its routine implementation in clinical practice [81]. However, MRD measured by NGS has been shown to predict future recurrence across various studies [80]. At present, the number of centers capable of performing MRD detection using NGS is limited, which poses a significant challenge to the advancement of this technique. Hence, this situation is expected to change in the coming years. The era of increasingly personalized medicine encourages us to combine multidisciplinary efforts, aiming for the routine measurement of MRD by NGS in clinical practice soon [80].

### 7.4 NGS and Treatment Response Prediction

Multigene sequencing technologies, particularly NGS, provide a crucial foundation for targeted therapy and precision oncology by identifying actionable genetic alterations that substantially improve clinical outcomes. NGS acts as a molecular guide, helping therapeutic decisions in the complex landscape of tumor genetic heterogeneity [82]. The integration of NGS with advances such as artificial intelligence and multidisciplinary Molecular Tumor Boards is essential to maximize its potential and ensure equitable access to genomic‐driven treatments [82].

Specifically in NHL, NGS has significantly advanced our understanding of tumor biology by identifying recurrent somatic mutations that serve as therapeutic targets, enabling inhibition of pathways responsible for drug resistance and uncontrolled tumor proliferation. For example, recognizing mutations such as MYD88 and CD79B allows clinicians to predict response to targeted agents like Ibrutinib in DLBCL [27, 77]. Furthermore, detecting mutations implicated in resistance mechanisms—including alterations in the SWI‐SNF chromatin remodeling complex—guides the optimization of treatment strategies through personalized medicine. Leveraging synergistic molecular targets identified via NGS leads to more effective, tailored therapies and improved management of tumor resistance, thereby enhancing patient outcomes [27].

## 8 Evaluating the Diagnostic Value of NGS in NHL

NGS enables comprehensive and fast detection of multiple gene mutations in NHL, supporting precise molecular subtyping and the identification of key signatures like FL52 [83]. NGS also allows for non‐invasive diagnosis and monitoring through ctDNA analysis, making disease classification and management more accurate and individualized compared to traditional methods [60]. In addition, WES, as a high‐throughput NGS approach that sequences all coding regions, offers the most comprehensive molecular assessment of lymphoma genetics. WES has been successfully applied to major lymphoma subtypes including DLBCL, BL, follicular lymphoma, mantle cell lymphoma, splenic marginal zone lymphoma, and PTCL [63]. Importantly, NGS findings have revealed recurrent somatic mutations in NHL that serve as potential therapeutic targets for overcoming drug resistance [27]. Despite these advantages, several challenges limit NGS's full diagnostic potential, especially in resource‐limited settings. Many studies, particularly in developing countries, have small sample sizes, limited patient diversity, and lack long‐term follow‐up, which restricts the generalizability of their findings. Additionally, infrastructural limitations such as inadequate laboratory facilities, shortage of trained personnel, and inconsistent funding pose significant barriers to widespread NGS adoption. Variability in sequencing protocols and bioinformatics pipelines further complicates cross‐study comparisons and standardization. Addressing these issues through strategic investments in infrastructure, workforce training, and the development of region‐specific guidelines is essential to maximize the diagnostic benefits of NGS and improve patient outcomes worldwide [24, 72, 84].

## 9 Challenges, Standardization, and Policy Pathways for Equitable NGS Implementation

### 9.1 Cost and Accessibility of NGS Technology

Translating NGS into equitable global practice for NHL requires addressing several interconnected barriers, especially in developing countries. These include financial accessibility, technical standardization, and infrastructural support, which often exacerbate one another. While diagnostic procedures may not constitute the most significant cost in cancer patient treatment, they are increasingly becoming central to clinical decision‐making. Optimizing these diagnostic procedures is crucial for two main reasons [1]: the steady rise in mutation‐driven treatments in recent years, and [2] the emergence of tumor‐agnostic therapies based on molecular alterations that span various tumor types [85]. Hence, the introduction of NGS has significantly enhanced the detection of mutations, profoundly impacting the diagnosis and treatment of various diseases. While diagnostic procedures account for a relatively small portion of total pathway costs, there is growing attention on the influence of NGS on budgeting and its cost‐effectiveness compared to standard single‐testing methods [86]. Recent literature highlights the cost‐effectiveness of NGS as a strategy for oncology biomarker testing, particularly under certain conditions [87]. However, developing countries face challenges such as limited human development and restricted spending on education and research, among various other constraints [24]. The primary challenge facing genomic researchers and clinicians is the scarcity of resources. Consequently, genomic tools, particularly genome sequencing technologies that are becoming essential, are not widely accessible. Also, establishing a genome sequencing facility equipped with NGS technology remains a significant hurdle, especially in developing regions compared to the developed world [24]. Hence, genome sequencing technologies have been restricted to institutions that can afford the substantial costs associated with establishing and operating genome sequencing facilities [24]. This financial inaccessibility is critically compounded by a lack of standardized and reproducible protocols, which further elevates the operational complexity and cost.

### 9.2 Standardization of NGS Protocols and Data Analysis

While diagnostic NGS has become increasingly prominent in clinical settings for evaluating somatic mutations in cancer, inadequate standardization of sequencing parameters remains a significant barrier to its widespread implementation in routine clinical practice [88], particularly for variants present at low allele frequencies [89]. Currently, innovative error correction strategies—both computational and experimental—are being developed to reduce the high error rates associated with diagnostic NGS [90]. To enhance standardization in diagnostic NGS, estimating the appropriate coverage depth is a recommended starting point for assessing thresholds related to specific NGS assays. However, there remains a lack of published guidance concerning minimum technical requirements and their reporting in NGS, which is particularly crucial for detecting clonal and subclonal mutations in cancer diagnostics. This gap is largely attributable to the wide variety of library preparation approaches and the numerous variables that influence each specific NGS assay, making standardization challenging, alongside inter‐laboratory variability. Therefore, establishing minimum technical requirements and standardized reporting in NGS is highly desirable [91]. On the other hand, the interpretation of NGS data presents significant challenges due to the genome's size and complexity, as well as the technical errors that can arise during sample preparation, sequencing, and analysis. These errors can be effectively understood and mitigated through the use of reference standards—well‐characterized genetic materials or synthetic spike‐in controls—that facilitate the calibration of NGS measurements and enhance the evaluation of diagnostic performance [92]. Therefore, genomic data manipulation and analysis tools are indispensable for advancing genomic research. Although many of these tools are available under various open‐source licenses, numerous advanced options are commercialized or require complex licensing procedures, often exceeding the financial resources of institutions in developing countries [24]. Nonetheless, the informed use of reference standards and associated statistical principles remains crucial for ensuring rigorous analysis of NGS data, which is essential for its future clinical application [92].

Building on these advancements, significant progress has been made in the classification of lymphoid neoplasms, driven by genomic studies and international collaboration. These efforts have leveraged robust analytical methods to refine diagnostic criteria, recognize new entities, and inform the proposed International Consensus Classification, ultimately enhancing clinical management [84].

Together, these developments highlight the transformative potential of accessible genomic tools and rigorous data analysis in improving healthcare outcomes worldwide.

Addressing these technical hurdles and the overarching financial constraints demands more than ad‐hoc solutions; it requires deliberate, integrated policy frameworks tailored to resource‐limited environments.

### 9.3 Toward Integrated Policy and Implementation Frameworks

In light of these challenges, effective policy frameworks and targeted implementation strategies are essential to bridge the access gap to NGS technologies in resource‐limited settings. The literature underscores that genomic research is a cornerstone for advancing health, drug development, and food security, necessitating that governments in developing countries prioritize its funding and policy support. Successful models, such as South Africa's experience with targeted national funding, demonstrate a viable pathway forward [24, 93]. While affordable, portable sequencing technologies (e.g., Oxford Nanopore's MinION) can lower initial infrastructure and expertise barriers, significant challenges persist. These include weak foundational infrastructure, limited computational resources, a lack of regulatory and ethical frameworks, and high ongoing costs that threaten laboratory sustainability [24]. Addressing this complex problem requires a coordinated approach. Countries should invest in core infrastructure, establish context‐appropriate standards and regulations, develop specialized training programs, and promote regional genomics centers and international collaborations [24].

Moreover, fostering international collaborations and continuous workforce development is vital to sustain and expand genomic capabilities in developing countries [94, 95]. Collectively, implementing such an integrated policy framework is crucial for translating the potential of genomics into tangible improvements in diagnostics and therapy, thereby contributing to the reduction of global health disparities in diseases like NHL.

## 10 Conclusion

Recent advances in medical genetics promise significant improvements in cancer diagnosis, treatment, and overcoming drug resistance, with particular benefits anticipated for patients with NHL. NGS stands out as a groundbreaking achievement in genetic science, still in its nascent stages, with the potential to play a pivotal role in the future diagnosis and treatment of this patient population.

Developed countries have already embraced NGS, maximizing its utility. In contrast, developing countries face challenges in adopting this method due to limited laboratory resources, inadequately equipped research facilities, and a dearth of experts in the field. Despite these obstacles, the progress of NGS in cancer diagnosis within these regions is undeniable. Notwithstanding the hurdles, the NGS method is expected to emerge as a cutting‐edge tool for both cancer diagnosis and the selection of targeted drugs in developing countries soon.

## Author Contributions

**Mohammadreza Saeidnia:** conceptualization, data curation, formal analysis, funding acquisition, investigation, methodology, project administration, resources, software, supervision, validation, visualization, writing – original draft, writing – review and editing. **Mehdi Shokri:** methodology, software. **Hassan Nourmohammadi:** conceptualization, investigation, methodology, project administration. **Safa Radmehr:** data curation, validation, writing – original draft, writing – review and editing. **Mashallah Babashahi:** investigation, writing – review and editing. **Mohamad Moradi:** data curation, resources. **Maryam Karimian:** conceptualization, data curation, formal analysis.

## Funding

The authors received no specific funding for this work.

## Ethics Statement

This study is a type of review study and does not need to approve the code of ethics and consent of the participants.

## Consent

All participants provided written consent for their data to be published in this article.

## Conflicts of Interest

The authors declare that they have no competing interests related to this study.

## Transparency Statement

The lead author Mohammadreza Saeidnia, Hassan Nourmohammadi affirms that this article is an honest, accurate, and transparent account of the study being reported; that no important aspects of the study have been omitted; and that any discrepancies from the study as planned (and, if relevant, registered) have been explained.

## Data Availability

The data sets generated and analyzed during the current study are available from the corresponding author upon reasonable request.
